# Letter from Vienna

**Published:** 1882-02

**Authors:** Wm. H. Fitch

**Affiliations:** Rockford, Ill.; Vienna


### Foreign Correspondence.

ARTICLE XI.

VIENNA, DEC. 26, 1881.

MESSRS. EDITORS:—Medical matters in this city seem very much the same to the writer as they did twelve years ago, when I attended lectures and private courses at the General Hospital for fourteen months. By some accident, I happened on my arrival in the city to meet a few American students at my hotel, who assured me everything was quite dull, and that I would see all of the city I wanted to in a few days, and that it did not come up to their expectations as a place for medical study. I found most of them to belong to a class of American students which is quite too numerous for our good name; who, while they enjoy the credit of completing their medical education abroad, spend more of their time at concerts, dance-houses and gaming-tables than at the hospital, and in many ways go contrary to the expectations of tender parents and anxious guardians. One young man was finding fault with the chambermaid about fleas in his bed. She told him he had brought them from the hospital. He replied it could not be so, as he had not been at the hospital for six weeks. Vienna holds out some attractions to a certain class of students more enticing than its medical advantages, and it is far better for the average young man to have practiced a few years, and become thoroughly convinced that he loves his profession, and to have found out his weak points before coming abroad. There are many industrious young men here, however, I am happy to say, who work early and late, and make the most of every opportunity. Oppolzer, Hebra, Dumreicher and Skoda, whom I listened to before, are dead, but supplanted by Bamberger, Albert, Kaposi and Schrötter, who fill the chairs with perhaps as much ability and acceptance.

Albert, the successor of Dumreicher and colleague of Billroth, although comparatively a young man, is very popular, and his clinics are more frequented, I should judge, than Billroth's. Both of them are poor speakers, much of their lectures being inaudible, while they are surrounded by such a crowd of assistants that it is almost impossible for an ordinary student, from his seat, to see anything of the progress of an operation of any importance, and the lucky one who sits inside the ring as guest for two weeks may reap more benefit than a regular attendant for a whole winter. I think this detail may not be without interest, as I know many are seriously disappointed at what they are able to see in these, as in some other departments.

They have here, as in some other places I have visited, a regulation that retires a professor when he has reached the age of sixty, which I think in some respects wise; which, although in some cases it deprives the student of the accumulated experience of age and years of teaching, relieves the authorities of the unpleasant responsibility of asking the resignation of some who have outlived their usefulness, of which the venerable professor is not always the best judge. When a man, like Prof. Arlt, instead of talking from his profound knowledge and extensive experience, sits down and reads his lecture from one of his not too recently printed volumes, it is time to retire; and I believe this is his last year.

As is well known by many, there are here professors ordinary and extraordinary; the one belonging to the faculty proper, and attendance upon whose lectures is obligatory upon the student intending to graduate, while the same is not required with the other; and consequently, while he may have the same clinical material, he has but few followers, and really offers far better opportunities for observation and study than the faculty professor. For instance, while Arlt's clinics are crowded, Yaeger has generally but half a dozen, and material abundant, and such is the case with some others, and while some of the regular faculty are clamoring for larger audience-rooms, new amphitheaters, and various other accommodations for their hearers, students are clamoring for more professors in some of the crowded departments, and better facilities to see, hear and learn. At present, the authorities seem to give but little heed to either petitioner.

Happening in to Prof. Carl Braun's obstetrical and gynæcological clinic, I heard the report of a patient who had died in six days after confinement, from fatty metamorphosis of heart and other organs. She had, in all, about fifty eclamptic convulsions. These came on before delivery, which was effected with forceps; without, however, influencing the spasms. She then had hot bath and purgatives, without effect. Then thirty grains of chloral were given, which checked them for two hours, when they recurred, and the dose was repeated until two drams had been taken without benefit, or only of a temporary character. Then she was bled several ounces, to have the blood tested for ammonium carb., when the convulsions ceased for several hours, and recurred. The examination did not reveal ammonium carb. The convulsions recurred, and these and other remedies were used, alone and in combination, but the pulse grew weaker and faster, until the patient expired. The lecturer remarked that the patient did not die of medicine, albuminuria, septicæmia, etc., but fatty metamorphosis. She had had the best of treatment known to the medical profession; and had the Almighty attended the case, He couldn't have saved her. The lecturer had given up venesection for many years, having no faith in its efficacy. Few patients recover who have convulsions before delivery.

Another patient was presented, upon whom an operation for recto-vaginal fistula had been performed without success. A second operation had been performed, with same unsuccessful result. He proposed to operate a third time, by first cutting from the vagina completely through the perineum, to the rectum, and make, as it were, a complete laceration, and also by cutting through the posterior part of rectum and sphincter; hoping by this method to secure a result, which by most careful manipulations he had failed to secure.

Such fistulas never occur in the wards of the hospital, or very seldom occur, and if they do, always heal themselves. It is only in aggravated, neglected and badly handled cases that an operation is necessary. As an illustration of the practice of the hospital in such cases, a patient was brought in who had been in labor for some hours, and in whom there had been no progress for an hour. Forceps were applied and, as laceration was threatened, the labia were cut on either side and delivery without rupture effected. The vagina was washed out with carbolic acid, the incisions closed with sutures and the vulva dusted with idoform. The patient was then carried back to the ward. I forgot to mention that this patient was somewhat œdematous, the urine contained albumen, and convulsions were feared, but none occurred.

The next patient was one with a deformed pelvis, upon whom craniotomy had once been performed and had been recommended in the event of a second pregnancy to return at the fourth month for artificial and premature delivery. This had been safely accomplished without any complication, and the patient, at the end of eight days, was ready to leave the hospital.

Much more attention is given to antiseptics in this department than when I was here before, as it was quite uncommon then to wash out the uterus or vagina with carbolic acid, and students gave far less attention to themselves as conveyors of infection than now. A preserved specimen, consisting of the neck of uterus and large epithelioma, which had been removed from a woman several years before, was passed around the class. The patient made a good recovery and became pregnant. The os was so hard and contracted from the cicatrix that premature delivery was induced. She was again pregnant; the os had been scarified a number of times and dilated, and now she was pregnant again; and the question arose as to whether she should be permitted to go to her full term of confinement. His experience had not been very gratifying with such cases; nevertheless, as the patient was quite anxious to properly terminate her pregnancy, and as the os was in a far more pliable and elastic condition from the treatment she had received, he was quite inclined to grant her request. This is but a sample, in brief detail, of what one sees and hears at his clinics daily, and gives even an inadequate idea of the advantages to be enjoyed in this department. Frequent allusions are made during these lectures to New York and Chicago men of prominence in these departments, while their obstetrical and gynæcological instruments are frequently shown and used.

Nevertheless, as is well known, it is not the teachers in this department we come here to see, as we have the best in the world at home. Give the student at home the material and the opportunity to use, as we already have in some branches, and he need not come to this far-off land to complete his education.

ROCKFORD, ILL. WM. H. FITCH, M.D.

---

THE MENTAL PHASE OF "COLD-CATCHING."—It is noteworthy as a curious yet easily explicable fact that few persons take cold who are not either self-consciously careful or fearful of the consequences of exposure. If the attention be wholly diverted from the existence of danger, by some supreme concentration of thought, as, for example, when escaping from a house on fire or plunging into cold water to save a life, the effects of "chill" are seldom experienced. This alone should serve to suggest that the influence exerted by colds falls on the nervous system. The immediate effects of a displacement of blood from the surface and its determination to the internal organs are not, as was once supposed, sufficient to produce the sort of congestion that issues in inflammation. If it were so an inflammatory condition would be the common characteristic of our bodily state. When the vascular system is healthy and that part of the nervous apparatus by which the caliber of the vessels is controlled performs its proper functions normally, any disturbance of equilibrium in the circulatory system which may have been produced by external cold will be quickly adjusted. It is therefore on the state of the nervous system that every thing depends, and it is, as we have said, on the nervous system the stress of a "chill" falls. Consciousness is *one* element in the production of a *cold*, and when that is wanting the phenomena is not very likely to ensue. It is in this way that persons who do not cultivate the fear of cold-catching are not as a rule subject to this infliction. This is one reason why the habit of wrapping up tends to create a morbid susceptibility. The mind by its fear begetting precaution keeps the nervous system on the alert for impressions of cold, and the centers are, so to say, panic-stricken when even a slight sensation occurs. Cold applied to the surface, even in the form of a gentle current of air somewhat lower in temperature than the skin, will produce the "feeling" of a "chill." Conversely a thought will often give rise to the "feeling" of cold applied to the surface—for example, of "cold water running down the back." Many of the sensations of cold or heat which are experienced by the hypersensitive have no external cause. They are purely ideational in their mode of origination, and ideal in fact.—*London Lancet.*

